# Biomarkers of Oxidative Stress in Metabolic Syndrome and Associated Diseases

**DOI:** 10.1155/2019/8267234

**Published:** 2019-05-05

**Authors:** Rosa Vona, Lucrezia Gambardella, Camilla Cittadini, Elisabetta Straface, Donatella Pietraforte

**Affiliations:** ^1^Biomarkers Unit, Center for Gender-Specific Medicine, Istituto Superiore di Sanità, Rome, Italy; ^2^Core Facilities, Istituto Superiore di Sanità, Rome, Italy

## Abstract

Metabolic syndrome (MS) represents worldwide public health issue characterized by a set of cardiovascular risk factors including obesity, diabetes, dyslipidemia, hypertension, and impaired glucose tolerance. The link between the MS and the associated diseases is represented by oxidative stress (OS) and by the intracellular redox imbalance, both caused by the persistence of chronic inflammatory conditions that characterize MS. The increase in oxidizing species formation in MS has been accepted as a major underlying mechanism for mitochondrial dysfunction, accumulation of protein and lipid oxidation products, and impairment of the antioxidant systems. These oxidative modifications are recognized as relevant OS biomarkers potentially able to (i) clarify the role of reactive oxygen and nitrogen species in the etiology of the MS, (ii) contribute to the diagnosis/evaluation of the disease's severity, and (iii) evaluate the utility of possible therapeutic strategies based on natural antioxidants. The antioxidant therapies indeed could be able to (i) counteract systemic as well as mitochondrial-derived OS, (ii) enhance the endogenous antioxidant defenses, (iii) alleviate MS symptoms, and (iv) prevent the complications linked to MS-derived cardiovascular diseases. The focus of this review is to summarize the current knowledge about the role of OS in the development of metabolic alterations characterizing MS, with particular regard to the occurrence of OS-correlated biomarkers, as well as to the use of therapeutic strategies based on natural antioxidants.

## 1. Introduction

Metabolic syndrome (MS) represents worldwide public health issue. It is characterized by a group of metabolic risk factors in the same person. The key factors are obesity, measured by waist circumference and body mass index (BMI), dyslipidemia, increased blood pressure, hyperglycemia, and insulin resistance [1].

A persistent increase of MS is a risk factor for type 2 diabetes (T2D), cardiovascular diseases (CVD), and premature mortality [1].

The pathogenesis of MS is very complex and not yet clear. Several studies support the concept that oxidant/antioxidant imbalance may play an important role in its manifestations [1, 2].

Increased biomarkers of oxidative stress (OS) and decreased antioxidant defenses have been measured in blood of patients with MS suggesting an *in vivo* overproduction of oxidizing species [1–6]. In particular, it has been reported that patients with MS have decreased antioxidant protection, in the form of depressed serum vitamin C and *α*-tocopherol concentrations, decreased superoxide dismutase (SOD) activity, and increased protein and lipid oxidation levels [1–6].

Many studies are focused on preventing OS in MS. Recent literature data suggest that diets that are rich in whole grain cereals, fruits, and vegetables, with low animal fat consumption, can ameliorate OS status [7, 8].

This review is aimed at presenting an overview on the role of OS in the pathogenesis of MS and the related diseases. In particular, it is focused on (i) mitochondrial redox state and dysfunction, (ii) most reported and validated biomarkers of stress in metabolic disease manifestations, and (iii) benefit of various nutritional antioxidants. In addition, the role of “gut microbiota” in MS will be described.

## 2. Oxidative Stress: An Overview

OS results from unbalanced production of reactive oxygen and nitrogen species (ROS and RNS, respectively) associated with decreased amount/expression and impaired activity of antioxidant systems. When available in appropriate low amounts, ROS and RNS act as signal transduction molecules driving cell activities and providing cell protection [9]. On the other hand, when produced in excess, as in the case of inflamed tissues, they can generate further highly reactive species able to oxidize irreversibly proteins, lipids, and nucleic acids. Very important is the oxidative modification of critical enzymes or regulatory sites, whose redox modification triggers cell signaling alteration and programmed cell death [9].

### 2.1. ROS and RNS in Physiology and Pathophysiology

A suitable equilibrium between ROS and RNS generation and antioxidant levels allows, without any sort of damage, either the cross talk between cells or the control of fundamental intracellular functions, such as cell-cell interactions, proliferation, differentiation, migration, and contraction. These activities are carried out through the direct or indirect reversible-redox modification of critical targets located within catalytic enzymes or regulatory sites [9]. These targets include iron-sulfur clusters, metals, flavin and nicotinamide cofactors, and quinones. The most relevant physiological targets are protein thiol/disulfides, with particular regard to those present within catalytic enzymes or regulatory sites, as well as in receptors, channels and transporters, transcription factors, kinases, and phosphatases. The reversible thiol oxidation products include sulfenic acid (sulfenylation) and the adducts with ^•^NO-derived species (S-nitrosylation), glutathione (S-glutathionylation), and H_2_S (S-sulfhydration). These oxidative modifications represent the finest regulation of redox-based signaling affecting protein activity, protein–protein interactions, and protein location, inducing conformational and functional changes of fundamental target macromolecules involved in redox signaling. A slight imbalance by oxidant formation can be counteracted by a performance activity of the antioxidant system, which allows cells to return to their physiological status. In pathological condition, such as inflammation, atherosclerosis, ischemia/reperfusion injury, and diabetes mellitus, the oxidant-generating enzymes in tissues are activated to produce higher amounts of ROS and RNS at nonphysiological locations. These amounts, in association with the impaired activity of the antioxidant systems, lead to the irreversible oxidation of proteins, lipids, and nucleic acids [9, 10]. This unbalanced redox equilibrium, called OS, triggers cell signaling alteration leading to loss of essential cellular functions, senescence, and programmed cell death [9, 10].

### 2.2. Sources of ROS and RNS

ROS and RNS are chemical heterogeneous molecules that include radical species, such as superoxide anion (O_2_^•^), hydroxyl radicals (^•^OH) and nitric oxide (^•^NO), and nonradical species, such as hydrogen peroxide (H_2_O_2_), hypochlorous acid (HClO), and peroxynitrite (ONOO-). They are continuously produced within the cells particularly as (i) the result of the electron transfer processes in the mitochondria and (ii) the activity of several inflammation-linked enzymes, such as NADPH oxidase, nitric oxide synthase (NOS), xanthine oxidase, myeloperoxidase (MPO), lipoxygenase (LOX), and cyclooxygenase (COX) [9]. Interestingly, O_2_^•^ and ^•^NO are poor oxidizing molecules, with behavior linked to their chemical structure and their thermodynamic properties. These characteristics are expressed by the standard one-electron reduction potential, a parameter suitable to predicting the hierarchy of radical reactivity in terms of ability to transfer the unpaired electron to any oxidized species [11]. The values range from positive one-electron reduction potential (highly oxidizing species) to negative one-electron reduction potential (highly reducing species) [11]. The one-electron reduction potential of O_2_^•^ is 0.94 V [11], while the one of ^•^NO is -0.80 V [12]. These values are by far lower than the ones taken for the most reactive unspecific ^•^OH (2.3 V) and confer to O_2_^•^ a low reactivity and to ^•^NO to be almost unreactive with most of the biological molecules. Notwithstanding their poor oxidizing potential, O_2_^•^ and ^•^NO represent the central nucleus of cellular oxidative chemistry, because they both directly and indirectly control the intracellular redox-based signaling and are the precursors of the most important oxidizing species produced under pathological conditions. Compared to many free radicals, O_2_^•^ has a relatively long half-life that permits diffusion inside the cell where it can be converted to H_2_O_2_ by the action of SOD. At low concentrations, H_2_O_2_ diffuses in intracellular compartments and reacts with specific protein residues, such as cysteine, methionine, and selenocysteine, assuming a key role in the regulation of the intracellular redox signaling [9]. At higher concentrations and in the presence of transition metals, H_2_O_2_ generates however the strong oxidant ^•^OH, which randomly reacts with all the most important macromolecules, inducing lipid peroxidation and oxidation of protein, DNA, and carbohydrates, leading to impairment of their activity and functions. ^•^NO is generated via enzymatic reaction catalyzed by the different isoforms of NOS. Moreover, ^•^NO can regulate intracellular signaling by directly inducing posttranscriptional reversible modification of cysteine residues in proteins, i.e., s-nitrosylation. On the other hand, the activation of intracellular pathways leading to ROS formation allows ^•^NO to rapidly react with O_2_^•^ and to produce the strong oxidant ONOO^−^ [1, 13]. The oxidative chemistry mediated by ONOO^−^ is led by its direct reactivity with several cellular targets (including CO_2_, hemoproteins, DNA, lipids, and protein residues such as thiols, tyrosine, tryptophan, and methionine) or indirect- CO_2_-dependent oxidations mediated by strong oxidizing radicals, such as ^•^NO_2_ (nitrogen dioxide radical) and carbonate radical (CO_3_^**•**^). Importantly, with the further increase of NOS activity such as that characterizing inflammatory conditions, ^•^NO_2_ rapidly reacts with ^•^NO forming dinitrogen trioxide (N_2_O_3_), which in turn mediates nitrosylation of target compounds, such as thiols. Interestingly, under inflammatory conditions the increased OS in tissues can lead to the consumption of substrates or to the oxidation of cofactors, in the specific case of (6R)-5,6,7,8-tetrahydro-L-biopterin essential for the correct NOS activity promoting O_2_^•^ instead of ^•^NO formation, referred to as endothelial NOS (eNOS) uncoupling. This condition increases further OS and decreases ^•^NO bioavailability leading to hypertension and vascular dysfunction in animal models of obesity, increasing further the risk of CVD diseases [13–15].

The prooxidant heme-containing MPO is released by activated neutrophils and monocytes/macrophages in tissues where it catalyzes the conversion of H_2_O_2_ to hypohalous acids (HOX; X=Cl, Br, and SCN) in the presence of halide and pseudo-halide ions [16]. The major reactive species produced by MPO under physiological conditions are hypochlorous acid (HOCl), hypothiocyanous acid (HOSCN), and species with nitrating activity in the presence of NO_2_^−^ and H_2_O_2_. These species are key contributors to the physiological microbicidal activity of phagocytes. However, excessive generation of MPO-derived oxidants has been linked to tissue damage in many diseases, especially in those characterized by acute or chronic inflammation [17].

#### 2.2.1. Mitochondrial Functions and OS

Mitochondria are crucial, multifunctional organelles, which actively regulate cellular homeostasis. Their main function is to produce energy as adenosine triphosphate (ATP) via citric cycle (tricarboxylic acid cycle, Krebs cycle). Other cell functions include ionic homeostasis, production and regulation of ROS, lipid and carbohydrate utilization, pH regulation, steroid hormone synthesis, calcium homeostasis, thermogenesis, and cell death [18–20].

As mentioned before, mitochondria are the primary intracellular site of oxygen consumption and the major source of ROS, most of them originating from the mitochondrial respiratory chain. Dysfunction of the respiratory chain may lower the ATP production, increase ROS production, reduce antioxidative capacity, and induce apoptosis. ROS, highly reactive molecules radicals, and nonradicals have the ability to capture electrons from molecules (proteins and nucleic acids) with whom they get in contact, leading to cell damage. In healthy cells, an intricate homeostatic system regulates and maintains optimal mitochondrial function. A failure of this system is observed in obesity, asthma, and metabolic syndrome progression [21]. Mitochondrial dysfunction, characterized by proapoptotic Bax and Bcl-xS proteins, reduces the expression of the antiapoptotic protein Bcl-xL and cytochrome C release and partially activates caspase cascade, high protein carbonyl content, and 8-hydroxy-2¢-deoxyguanosine. Growing evidence suggests that these dysfunctions have a strong relationship with various MS components, resulting in clinical complications [22, 23]. Cheng and Almeida declare that mitochondrial dysfunction is an early pathophysiological event in insulin resistance and obesity development [24]. Mitochondrial dysfunction leads to the activation of stress pathways, which reduce cellular sensitivity to insulin, limiting nutrient influxes and preventing further damages. Chronically, in organs such as liver and skeletal muscle, it appears as reduced mitochondrial metabolism and insulin resistance, following consequent hyperinsulinemia and different nutrient storages in adipose tissue [25–27]. In addition to this, in epithelial and vascular endothelial cells, mitochondrial dysfunction interferes with •NO synthesis leading to oxide/nitrate stress.

Several studies demonstrate that in different tissues, mitochondria adapt physically to nutrient availability and that obesity causes mitochondrial OS and dysfunction [28, 29]. O'Keefe and Bell proved that, apparently, high caloric intake raises plasma free fatty acid (FFA) and glucose levels, which are closely associated with high ROS generation and obesity exacerbation [1, 30]. Conversely, calorie restriction alleviates sarcopenia [31].

In cardiomyocytes of young patients, an excess of body weight impairs mitochondrial function also in the absence of heart failure and diabetes [32]. Moreover, in humans and mouse models, obesity results in mitochondrial dysfunction, skeletal muscle, and adipose tissue [33].

Mitochondria are subject to dynamic processes in order to establish a control system related to survival or cell death and adaptation to changes in the metabolic environment of cells. The mitochondrial dynamic includes several processes such as fusion and fission, biogenesis, and mitophagy. Modifications of the mitochondrial dynamic in organs involved in energy metabolism such as the pancreas, liver, skeletal muscle, and white adipose tissue could be of relevance for the development of insulin resistance, obesity, and type 2 diabetes. Metabolic status can condition the number, form, and function of mitochondria, influencing organ function. Conversely, changes in mitochondrial dynamic influence organ metabolism. Mitochondrial biogenesis is critical for the normal function of cells, and it can be produced in response to an oxidative stimulus. Mitochondrial fusion is linked to increased ATP production, while the inhibition of this process is associated with ROS production [34–36].

### 2.3. Detection of ROS and RNS in the Biological System

To characterize ROS/RNS and to understand their role in the mechanisms leading to the different pathological conditions and diseases, the specific detection and quantification of the oxidants produced are fundamental. Luminescence-based techniques, such as fluorescence, chemiluminescence, and bioluminescence, have been applied to biological systems (cell-free systems, *in vitro* cell cultures, and *in vivo* animal models) to detect directly cellular oxidizing species, such as O_2_^•^, H_2_O_2_, ^•^NO, and ONOO^−^. These methods rely to the use of specific probes, such as dihydrorhodamine, dihydroethidium, dihydrodichlorofluorescein, Amplex Red, and boronates. All of these are able to interact with the formed oxidants and to provide information about the different species produced in the studied system. Initially, these methods have been used to study the changes of the redox environment in cell systems. Subsequently, with increasing knowledge on chemical reactivity and suitable/useless end product formation, several limitations on the use of these probes have been proved [37–39]. These limitations deal with the chemical features of the probes as well as their reactivity, such as (i) secondary chemical interactions between probe end products, the probe-derived radicals, and the oxidants generated in the studied system; (ii) redox cycling and generation of ROS by probes and their oxidation products; (iii) probe interaction with metals and with the intracellular antioxidants; (iv) intracellular compartmentalization of probes and sites of oxidant formation; and (v) probe sensitivity to light and pH changes [37–39]. Another method suitable for ROS and RNS identification in biological systems is the electron paramagnetic resonance spectroscopy (EPR) that, in combination with the spin trapping technique, allows identifying oxidants such as O_2_^•^, ^•^OH, and ^•^NO. The spin trapping technique permits the species-specific and time-dependent detection of products, showing typical EPR spectral characteristics. The signals obtained from the different formed radicals may be compared to complementary simple control experiments using inhibitors/scavenger/competitor compounds. The main restrictions to the application of the EPR-spin trapping technique are (i) low rate/concentration of oxidant formation in the biological system, (ii) low trapping rate constant of the used probes, and (iii) reduction of the EPR-active spin adduct to EPR silent product(s) by endogenous antioxidants/reducing systems [40].

Recent literature data on this topic illustrate that the simultaneous use of different techniques, i.e., EPR, HPLC, fluorescence, and LC-MS, is the best way to identify ROS and RNS in biological systems. Each of them provides useful information concurring to identify the formed species by detecting the specific adducts and end products [38–40].

### 2.4. Tissue Antioxidant Systems

Cells contain both enzymatic and nonenzymatic antioxidants that work together to regulate ROS and RNS concentrations. The primary antioxidant enzymes in tissues that are able to detoxify directly oxidizing species include SOD, glutathione peroxidase (Gpx), and catalase (CAT), and bilirubin. Other fundamental antioxidant enzymes such as glutathione reductase (GR), peroxiredoxins (Prx), thioredoxin (Trx), thioredoxin reductase (TrxR), and glutaredoxins (Grx) also contribute to cellular protection by reducing oxidized critical thiols in key enzymes/proteins and maintaining the suitable intracellular redox state [1, 9, 41]. The nonenzymatic system includes endogenous compounds, such as reduced glutathione (GSH), uric acid, bilirubin, and ceruloplasmin, as well as dietary low-molecular-weight compounds, such as vitamins (vitamins A, C, and E), *β*-carotene, flavonoids, polyphenols, and zinc and selenium. The latter are the key components of enzymes such as Gpx and TrxR [1, 9, 41]. SODs are a group of metalloenzymes that can be compartmentalized in different cellular districts, such as cytosol (CuZn-SOD), mitochondria (Mn-SOD), and the extracellular matrix (EC-SOD). The main activity of this enzyme is to catalyze the conversion of O_2_^•^ to H_2_O_2_.

SODs then contribute to control ROS intracellular concentration and, in conjunction with CAT and GPx, to maintain the flux of H_2_O_2_ suitable for redox regulation of intracellular signaling. GPx is an enzyme dependent on the micronutrient selenium and plays a crucial role in the reduction of lipid oxidation and peroxide detoxification. Moreover, together with GR, it plays a critical role in GSH metabolism, reducing glutathione disulfide (GSSG) to GSH, by the NADPH-dependent mechanism. Prxs are thiol-specific antioxidant enzymes that reduce various cellular peroxide substrates, including H_2_O_2_ and ONOO-, using cysteine-containing active sites. TrxR and Grx have catalytic-redox-active cysteines and catalyze the reduction of protein mixed disulfides. Trx in particular has function in DNA and protein repair by reducing ribonucleotide reductase as well as methionine sulfoxide reductases.

Some of these antioxidants, proteins, and redox enzymes belong to a network connected through substrates and products. Together with oxidized/reduced glutathione and oxidized/reduced Trx, the redox couple NADP^+^/NADPH is crucial for the intracellular redox homeostasis. Indeed, it provides reducing equivalents for the two main detoxifying enzyme systems (GR/GPx/GSH and TrxR/Trx/Prx) involved in H_2_O_2_ removal, in maintaining the suitable thiol/disulfide intracellular equilibrium linked to redox signaling (sulfenic acids, S-nitrosothiols, S-glutathionylation, etc.) [9]. NADP^+^/NADPH and the redox enzymes also have a key role in controlling smooth muscle vascular relaxation through the regulation of the intracellular calcium concentration from the potassium channels, the sarco(endo)plasmic reticulum calcium ATPase (SERCA) pump, ion channels controlling membrane potential, and contractile-enhancing systems [42]. The main source of intracellular NADPH is the oxidative branch of the pentose phosphate pathway, with the cofactor being produced by the rate-limiting glucose-6-phosphate dehydrogenase and 6-phosphogluconate dehydrogenase. Other sources of NADPH are the cytoplasmatic and mitochondrial isoforms of isocitrate dehydrogenases, formyltetrahydrofolate dehydrogenases, methylenetetrahydrofolate reductase, and the NADP-dependent malic enzymes [43].

Low-molecular-weight compounds are fundamental antioxidant molecules because they can directly detoxify ROS and RNS and repair oxidized biological targets [1, 9, 41]. GSH is the predominant non-protein low-molecular-weight compound (0.5–10 mM) in animal cells and is the major cellular redox buffer, so that its concentration and the one GSSG are commonly used to calculate the GSH/GSSG ratio, universally used as an indicator of the cellular redox environment [9, 11, 23]. GSH reacts with several oxidizing species, including ^•^OH, HClO, and ONOO^−^, and is the substrate of GPx in its peroxide detoxification activity.

Vitamin C or ascorbic acid, the first antioxidant defense in tissues, is a biologically ubiquitous and water-soluble antioxidant able to donate an electron to potentially damaging oxidizing radicals such as ^•^OH, alkoxyl radical (RO^•^), peroxyl radical (ROO^•^), thiyl radical (GS•), and tocopheroxyl radicals (TO^•^). It has been shown that physiological concentrations of ascorbic acid inhibit oxidation of LDL, lipid, and protein. An important feature of ascorbic acid is acting synergistically with vitamin E and protecting this vitamin from the oxidative modifications. Vitamin E is indeed lipid-soluble, it is the primary antioxidant in LDL and lipid membrane, and its one-electron oxidation product, the *α*-tocoperoxyl radical, can be reduced by ascorbic acid. There are eight naturally occurring vitamin E isoforms, namely, *α*-, *β*-, *γ*-, and *δ*-tocopherol and *α*-, *β*-, *γ*-, and *δ*-tocotrienol. *α*-Tocopherol acts as a radical-scavenging antioxidant against lipid peroxidation by scavenging lipid-centered ROO^**•**^ before it attacks lipid in other molecules to yield lipid hydroperoxide (LOOH), and a lipid radical (L^•^), which propagates the chain oxidation. *β*-Carotene is a powerful singlet oxygen quencher and exhibits additional, strong antioxidant properties. It is very reactive to peroxyl radicals and DNA damage, but less so to ^•^OH and O_2_^•^.

Uric acid is synthesized mainly in the liver, intestines, and the vascular endothelium as the end product of an exogenous pool of purines, is a strong ROS and RNS scavenger (such as ^•^OH, ONOO^−^, and HClO), and has metal-chelating properties in blood plasma as in saliva.

**Figure 1:**
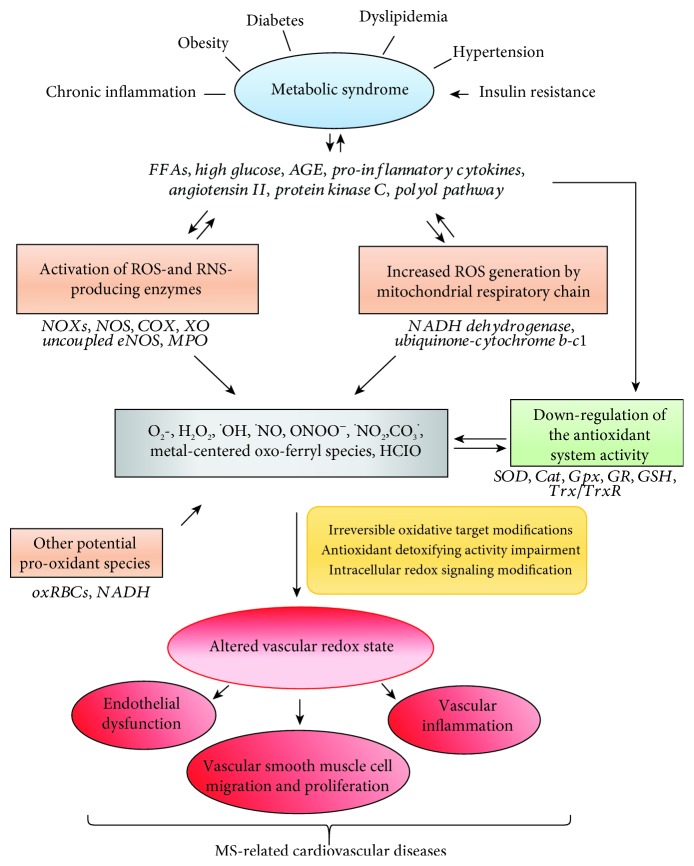
Oxidative stress and vascular implications in metabolic syndrome. Metabolic syndrome (MS) is characterized by risk factors having tissue oxidative stress (OS) as hallmark. Indeed, they are characterized by increased release and accumulation of proinflammatory mediators, such as free fatty acids (FFAs), high glucose levels, advanced glycation end-products (AGE), cytokines, and angiotensin II, as well as to the activation proinflammatory signals linked to the stimulation of protein kinase C (PKC) and polyol pathways. These conditions boost the increase in reactive oxygen (ROS) and nitrogen species (RNS) formation in tissues and in the vasculature through the activation of the related producing enzymes in the cytosol as well as in the mitochondria. The cytosolic enzymes include the different isoforms of NADPH oxidase (NOXs), nitric oxide synthase (NOS), cyclooxygenase (COX), xanthine oxidase, protein kinase C (PKC), uncoupled endothelial NOS (eNOS), and myeloperoxidase (MPO). Other potential sources of ROS and RNS are the oxidized RBCs (oxRBCs) and the increased NADH amounts. The former, forming in the vasculature under significant OS conditions, behave as prooxidant cells able also to release oxidant species. The latter, increased at expenses of NADPH under hyperglycemic conditions, can induce mitochondrial deregulation and ROS formation. Noteworthy for the MS-associated cardiovascular complications is the reduction in ^•^NO bioavailability in the vasculature, notwithstanding the NOS upregulation. Indeed, the simultaneous increase in the concentration of ^•^NO and O_2_^•^ allows these radicals to react fast generating the strong oxidant peroxynitrite (ONOO^−^), which deeply affects intracellular redox chemistry. The MS-associated diseases are also characterized by the downregulation of the antioxidant systems, including the depletion of GSH concentration and the decrease of the activity of the detoxifying enzymes, such as superoxide dismutase (SOD), catalase (Cat), glutathione peroxidase (Gpx), glutathione reductase (GR), and the couple constituted by thioredoxin (Trx) and thioredoxin reductase (TrxR). In addition, as in a vicious cycle, the increased ROS and RNS formation can further worse MS-related diseases by affecting in turn the intracellular pathway generating the proinflammatory mediators, as well as decrease the activity of the antioxidant systems. All these conditions result in the irreversible accumulation of oxidation products in proteins, lipids, and sugars, which allow the impairment of intracellular redox signaling and detrimentally affect vascular biology by promoting vascular inflammation, endothelial dysfunction, and vascular remodeling. These alterations underlie the typical MS-associated cardiovascular complications, such as coronary atherosclerotic disease, arterial hypertension, left ventricular hypertrophy, diastolic dysfunction, coronary microvascular disease, and autonomic dysfunction.

Bilirubin has been proved to be a powerful antioxidant in human blood plasma both in its unconjugated form and complexed with serum albumin. In addition to its ability to inhibit membrane lipid peroxidation acting in synergy with the membrane-bound *α*-tocopherol, bilirubin can scavenge both ROS and RNS and protect proteins from ROS- and RNS-mediated oxidative damage [41]. Prooxidant and antioxidant systems are shown in Figure 1.

### 2.5. Biomarkers of OS Damage

In addition to the indirect measurement of ROS and RNS levels, oxidized molecules reflect the damage mediated by OS in cells and tissues, and their measurement can be indicative for the occurrence of OS in a specific disease, as well as the potential efficacy of clinical treatments.

As already mentioned, ROS and RNS generically can react with all the macromolecules of biological importance in cell and tissues, generating oxidative modification in lipids, DNA, and proteins that, in some cases, can be the footprint of the oxidant generated [44, 45].

Most of these oxidized molecules can be measured in plasma by using ELISA kits or by HPLC.

Polyunsaturated fatty acids (PUFAs), in particular linoleic and arachidonic acid, are important targets of lipid peroxidation. Malondialdehyde (MDA) and 4-hydroxy-2-nonenal (HNE) are the most investigated end products of lipid oxidation. MDA is one of several low-molecular-weight end products formed via the decomposition of certain primary and secondary lipid peroxidation products. It is a specific marker of omega-3 and omega-6 fatty acid peroxidation [46]. 4-HNE is an *α*, *β*-unsaturated aldehyde that derives from the oxidation of essentially arachidonic and linoleic acid, which are the two most represented fatty acids in biomembranes [47]. It is toxic at high concentrations (from 1 *μ*M to 10 *μ*M), while it plays a role in signaling activities at lower concentrations (under 1 *μ*M) [47].

ROS and RNS generate a large number of oxidative modifications in DNA including nucleotide oxidation, strand breakage, loss of bases, and adduct formation. The ^•^OH radical can react with all purine and pyrimidine bases, as well as deoxyribose backbone, generating various products, the most common one being 7,8-dihydroxy-8-oxo-24′-deoxyguanosine (8oxodG), which can be assessed noninvasively in the urine [48–49].

The main cellular targets of ROS and RNS in tissues are doubtless the highly concentrated proteins, which undergo posttranscriptional oxidative modifications (oxidation, carbonylation, nitrosylation, and nitration) of specific amino acid residues (cysteine, aromatic amino acids, histidine, and methionine). Some of these modifications are reversible (i.e., some oxidation products and nitrosylation of thiol groups), while others are irreversible (carbonylation and nitration) and can lead to the alteration of protein expression and activity. The reversible oxidative modifications are involved in the physiologic redox regulation of cellular signaling and are involved in particular cysteine in specific proteins or enzymes. Cysteine oxidation usually starts with the formation of sulfenic acid (-SOH), disulfide bridges (S-S-), S-glutathionylation (protein-SSG), and S-nitrosylation (-SNO) [10, 50]. The reversibility is linked to the activity of low-molecular-weight antioxidants, such as GSH and vitamin C, as well as the activity of antioxidant enzyme, such as GR, Grx, Trx/TrdR, and Prdx. Protein S-nitrosylation (addiction of -NO group) can be induced by RNS, such as ^•^NO, nitroxyl, and ONOO^−^. This thiol modification, and its reversibility, plays a pivotal role in cell physiology and signaling, so that S-nitrosylation has been regarded as functionally equivalent to protein phosphorylation and dephosphorylation [50].

The irreversible oxidative protein modification can allow to the definitive alteration of protein expression and activity, which inevitably reflects on cellular trafficking and redox signaling. This is particular true for cysteine residues in specific proteins or enzymes in which the oxidation processes, going further sulfenic acid formation, proceeds with the formation of sulfinic (-SOOH) and sulfonic (-SOOOH) acids, leading to the irreversible inactivation of their activity [9]. The carbonyl groups can be generated after the attack of the ^•^OH radical against the residues of proline, lysine, and arginine [51–53]. Carbamylated proteins are interesting to use as biomarkers, because they may quantitatively reflect the burden of pathological conditions (inflammation and uremia) and are present in plasma or whole blood. Finally, protein tyrosine residues can undergo nitration reaction (addiction of -NO_2_ group) to form 3-nitrotyrosine and can be generated through several pathways including their selective reaction with ONOO- and the derived strong oxidants, such as ^•^OH, ^•^CO_3_, and NO_2_^•^, with nitrite anion at acidic pH, or with activated peroxidases in the presence of nitrite anion [52]. Protein nitration induces a loss and gain of protein function, compete with protein phosphorylation, stimulate the autoimmune response, and affect protein turnover. Biomarkers of OS are shown in Table 1.

## 3. Oxidative Stress in Diabetes and Metabolic Syndrome: State of the Art

Elevated OS in individual with T2D and MS has been shown to be one of the major risk factors for an increased risk of cardiovascular disease [1, 2, 5, 53]. T2D is a metabolic disease associated with increased formation of ROS and RNS, as well as decreased antioxidant potential [54]. The precise mechanism by which OS may accelerate the development of complications in diabetes is partly known [1]. T2D is characterized by chronically elevated blood glucose levels, which may be caused by increased insulin resistance and glucose intolerance. Persistent hyperglycemia in T2D causes nonenzymatic protein glycation and oxidative degeneration. In the early stage of protein glycation, the aldehyde group of sugar reacts with amino acids to produce Schiff's base, which undergoes a series of modifications to form Amadori rearrangement products [55]. The accumulation of advanced glycation end products (AGE) contributes to diabetic complications through direct tissue damage and activation of specific AGE receptors (RAGE) [56]. AGEs and RAGE interaction elicit OS generation in various types of cells and subsequently evoke proliferative, inflammatory, and thrombogenic reactions, playing an important role in the development and progression of diabetes-associated disorders [57]. Biomarkers of protein glycation in T2D are glycated hemoglobin (HbA1c) and glycated low-density lipoprotein (gl-LDL). HbA1c is more negatively charged than hemoglobin and has a higher oxygen affinity therefore reducing gaseous exchange to tissues. Glycated LDL loses its affinity for the LDL receptor, but it is not very inflammatory. Biomarker of oxidative degeneration in T2D is an oxidized low-density lipoprotein (ox-LDL) that, in contrast to gl-LDL, is a proinflammatory and proatherogenic particle containing protein adducts and inflammatory lipids that promote atherosclerosis. Studies have shown that ox-LDL within the vascular endothelium leads to the expression of monocyte chemoattractant protein-1 (MCP-1), known to promote vascular endothelial dysfunction and to increase thrombogenicity [58]. Increased amounts of ox-LDL, carbonylation of cellular proteins, and NADPH oxidase activity can occur in MS leading to enhance ROS formation, which indicates increased risk of atherosclerosis and myocardial infarction as well as increased OS in MS patients [58, 59]. Vascular endothelial dysfunction results in increased ROS production and decreased bioavailability of ^•^NO due to the formation of ONOO^−^-derived species with oxidizing/nitrating activity. Moreover, ONOO^−^ has been reported to inactivate prostacyclin synthase leading to the accumulation of inflammatory and prothrombotic eicosanoids [60, 61]. Finally, in endothelial cells, both gl-LDL and ox-LDL drive mitochondrial dysfunction and high levels of ROS production, especially of O_2_^•^ [62, 63].

MS, characterized by impaired glucose metabolism, dyslipidemia hypertension, and abdominal obesity, is frequently associated with T2D. As for diabetes, OS plays a pivotal role in the pathogenesis of the MS and in the progression of its complications. In MS, ROS production could be increased by an accumulation of plasma FFAs [64] that increases ROS and RNS formation and release in endothelial and vascular smooth muscle cells [64, 65]. FFAs constitute a trigger for ROS increase in adipose tissue, by stimulating NOX and lowering the activity of the antioxidant enzymes [5]. The exposure of adipose tissues to OS results in the development of systemic inflammatory state, which contributes to obesity-associated vasculopathy and cardiovascular risk [66].

Currently, hypertension is considered a primary risk factor for MS linked to the decreased ^•^NO bioavailability and/or to the FFA-mediated ROS increase in blood plasma. This link has been investigated in animal models of hypertension, in which ROS production, endothelial dysfunction, and lipid peroxidation markers have been found increased as a consequence of fructose administration [1]. The role of OS in hypertension has been proved also by the positive effects on ROS production and blood pressure measured after the antioxidant administration in animal models of hypertension.

**Figure 2:**
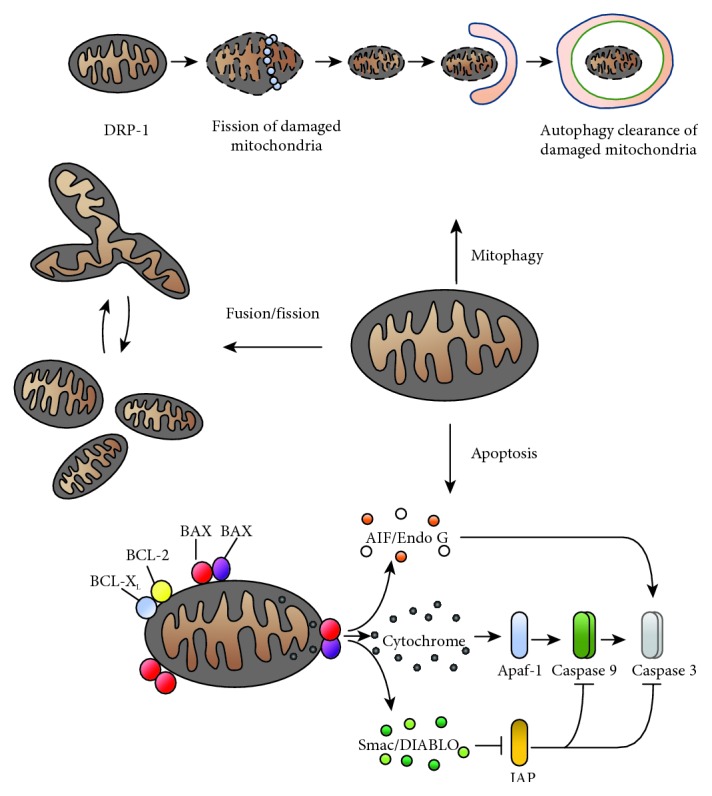
Mitochondria dynamics. Summary of major molecular events related to the mitochondria: apoptosis, mitophagy, and process of fusion/fission. In the apoptotic process, the function of BCL-2 family proteins in the control of integrity mitochondrial outer membrane is shown. In particular, the proapoptotic proteins BAX and BAK heterodimering open a channels, which allows the release of proteins such as the cytochrome-c, Smac/DIABLO, Endo G/AIF. These latter, in turn, led to caspase cascade activation, which induces the apoptotic modifications.

A number of studies, in human's as well as in animal's models, have stated that most diseases associated with MS, such as obesity, insulin resistance, hypertension, dyslipidemia, and diabetes, cause mitochondrial OS, altered mitochondrial morphology, and oxidative phosphorylation functions, as well as the activation of mechanisms leading to the induction of mitophagy and apoptosis (Figure 2) [1]. In particular, the accumulation of free cholesterol, ox-LDL, and glycated HDL has been reported to mediate endothelial dysfunction through the ROS-mediated impairment of mitochondrial functions and adipocytokines release boosting the atherogenic processes [5, 67–70]. Increased protein carbonyls and lipid peroxidation markers together with decreased activities of mitochondrial antioxidant enzymes (SOD and Gpx) have been measured in adipose tissues from obese patients [71].

Furthermore, the MS-linked pathological conditions, as mentioned before, have been reported to be linked to chronic inflammation mediated by hyperglycemia, FFA and AGE accumulation, and systemic insulin resistance [1, 72]. Several proinflammatory mediators, known to promote OS, are released in the vasculature where they boost endothelial activation and dysfunction, causing morbidity and mortality in a large number of MS patients. Among these mediators, inflammatory cytokines (leptin, tumor necrosis factor, and interleukin-6) [73] and high amounts of glucose [74] and FFAs [75] have been recognized to play a pivotal role in the deregulation of the activity of ROS- and RNS-producing systems, such as NADPH oxidases, NOS, and mitochondrial oxidases [1, 76].

The following paragraphs will analyze in detail the contribution of each oxidant source to OS occurrence in MS.

### 3.1. NADPH Oxidases in MS

NADPH oxidases are multi-subunit enzymes present in seven isoforms, called NOX 1–5, and dual oxidase DUOX proteins 1–2 [76]. These enzymes are expressed in a wide variety of cell types, including endothelial cells and vascular smooth muscle cells, in which the isoforms NOX2/NOX4 and Nox1/NOX4, respectively, are mainly expressed [76].

The increase in NOXs mediated by O_2_^•^ production has been detected in several MS-related diseases [1, 71, 76, 77]. In an animal model of mice overexpressing one of the NOX subunits (p22phox), an increase in vascular ROS concentration was found. Interestingly, in this study it was observed that a nutrition with a high-fat regimen induced the occurrence of inflammatory status and adipogenesis favoring obesity and MS phenotype appearance [78, 79]. Moreover, it has been found that FFAs and hyperglycemia promote protein kinase C activation. The activation of this protein is involved in OS-mediated endothelial dysfunction by promoting NOX phosphorylation [77, 79], by activating mitochondrial oxidases [78, 80] as well as the signaling linked to the MAPK p38-mediated NOX induction through endothelin-1 signaling [81, 82]. FFAs and glucose accumulation have been shown to enhance in endothelial and vascular smooth muscle cells, kidney cells, and adipose tissue, lowering in addition the antioxidant enzyme activity [5, 83]. Interestingly, in the endothelium of diabetic mice, FFA-mediated increase in O_2_^•-^ production has been reversed by the specific NOX inhibitor, diphenyleneiodonium [84].

Hyperglycemia has been found to promote the NOX-dependent increase in ROS formation in vascular smooth muscle cells, endothelial cells, and cardiomyocytes [78, 85, 86]. High glucose concentration promotes NOX activation through the stimulation of pathways linked to the protein kinase C and the Ca^2+^/calmodulin-dependent protein kinase and/or in the presence of AGE as well as of angiotensin II [1, 72, 78, 87].

The angiotensin II-mediated protein kinase C-dependent NOX activation has been reported to induce mitochondrial dysfunction in cardiomyocytes and endothelial cells [87, 88]. In addition, high glucose can also stimulate ROS production by inducing mitochondrial dysregulation through the alteration of internal membrane potential and the increase in the flux of electron transfer donors (NADH and FADH) into the mitochondrial respiratory chain [78]. Despite its involvement in hypertension onset, angiotensin II has been found to inhibit the insulin activity by its binding to the angiotensin type 1 receptor [89], which, in addition to NOX activation, contributes to the occurrence of vascular insulin resistance, endothelial dysfunction, apoptosis, and inflammation. Finally, hyperglycemia could modify cell signaling and antioxidant defense through the activation of the polyol sugar oxidation pathway, which reduces the intracellular amount of NADPH [90]. This cofactor is indeed essential for the activity of several enzymes (GR and TrxR) fundamental for the maintenance of the suitable intracellular GSH concentration and redox balance [9, 10]. In human endothelial cells, AGE, formed by the nonenzymatic reaction of reducing sugars with proteins, lipids, and nucleic acids, promoted NOX activation [91]. Interestingly, the administration of RAGE to diabetic mice partially counterbalances vascular dilation and inhibits NOX expression, confirming the key role of AGE/RAGE signaling in regulating OS and endothelial dysfunction in diabetes [92].

### 3.2. NOSs in MS

The major sources of ^•^NO in the heart tissue are (i) endothelial NOS (eNOS), mainly expressed in the coronary and cardiac endothelium; (ii) neuronal NOS (nNOS), mainly located in the cardiac myocytes [93]; and (iii) inducible enzyme isoform (iNOS), expressed in cardiac myocytes or in neutrophils migrated in this tissue under inflammatory conditions [94]. Among these isoforms, eNOS and iNOS expression was found increased in the heart of diabetic animals [95]. In agreement, by EPR spectroscopy, other authors in blood measured increased amounts of ^•^NO and augmented levels of some ^•^NO-derived oxidation products (lipid peroxidation, 3-nitrotyrosine formation, and nitrite and nitrate concentration) [94]. Notwithstanding the evidences highlighting its activation, in MS-related disease ^•^NO biological activities are in general downregulated due to a dysfunction of the radical metabolism and availability. As reported above, the activation of NOXs measured in the MS-related diseases could lead to increased ROS production. These may decrease •NO availability by (i) forming, through the fast direct reaction between ^•^NO and O_2_^•^ and the vasoconstrictor and cytotoxic oxidant ONOO^−^; (ii) eNOS uncoupling, which can produce O_2_^•^, instead of ^•^NO, under OS-mediated depletion of the enzyme cofactors, such as (6R)-5,6,7,8-tetrahydro-L-biopterin; and (iii) the NF-*κ*B-mediated release of proinflammatory cytokines (IL-6 and TNF-*α*) able to inhibit NOS activity [72, 94]. This is the case of dyslipidemia, hyperglycemia, insulin resistance, and hypertension, which affect NOS activity through ROS formation increase mediated by several sources (NOXs and xanthine oxidase, dysregulated mitochondria, and uncoupled NOS) activated by FFAs, high glucose, AGE, angiotensin II, etc. Other intracellular pathways have been reported to alter ^•^NO formation in MS by upregulating or downregulating the different NOS isoforms. In particular, in the myocardium, the activation of the leptin/STAT3 pathway upregulates the gene for iNOS, while that linked to leptin/JAK2/IRS-1 stimulates the Akt-mediated eNOS activity. A reduction in NOS activity has been found (i) as a consequence of the modification (OGlcNAcylation) of eNOS protein at the Akt site (condition favoring endothelial dysfunction in diabetic vascular complications); (ii) in hyperocholesterolemia, which impaired the signaling linked to soluble guanylate cyclase (the key enzyme of the •NO signaling pathway); (iii) following PKC activation, which results in the reduction of eNOS activity linked to the phosphorylation of Thr495, which contrarily to the phosphorylation at Ser1177 did not increase the enzyme activity; and (iv) following interaction between AGE and their soluble receptor, which in endothelial cells decreases both eNOS expression and activity [94].

### 3.3. Mitochondrial Oxidases in MS

The mitochondrial respiratory chain, specifically complexes I (NADH dehydrogenase) and III (ubiquinone-cytochrome b-c1), is the main source of intracellular O_2_^•^. Hyperglycemia and obesity allowed mitochondrial dysfunction, including ROS formation, impairing physiological respiration and decreased ATP production in the heart of animal models [96]. Hyperglycemic-linked mitochondrial dysfunction has been linked to the downregulated expression of the peroxisome proliferator-activated receptor gamma coactivator 1a (PGC-1a), a key factor for mitochondrial metabolism involved in oxidative phosphorylation [97]. FFAs, such as ox-LDL, can be the cause of mitochondrial dysfunction and/or apoptosis by impairing oxidative phosphorylation and ROS production in vascular endothelial cells and in cardiomyocytes [98, 99]. AGE and protein kinase C induce mitochondrial ROS formation in through NOX activation [76, 99, 100].

### 3.4. Red Blood Cells as Potential ROS and RNS Sources in MS

In addition to the biomarkers listed above, there are experimental studies that suggest red blood cells (RBCs) as possible gender-associated biomarkers implicated in the progression of MS [101].

RBCs, under physiological conditions, exert a scavenging activity towards reactive oxygen and nitrogen species often overproduced in morbidity states, for example, in inflamed tissues. Their deformability is an important prerequisite for vascular “antioxidant” functions. Conversely, under conditions of systemic OS, RBCs have an altered redox state and consequently a loss of their structural and functional characteristics, becoming in turn a source of reactive species and contributing to vascular damage.

A significant increase in RBCs displaying morphological alterations has been detected in patients with MS. In particular, differences in terms of cell aging, cell adhesion, and/or aggregation have been found. These data are in line with other literature data indicating erythrocytes as possible biomarkers of vascular disease [102].

Possible implications of OS in the integrity and function of RBCs have been also found in patients with non-insulin-dependent diabetes [103]. In particular, it has been demonstrated that in these patients, RBCs show ultrastructural alterations that could be countered by treatment with the antioxidizing drug *N*-acetylcysteine [103].

Importantly, altered RBC could represent a biomarker playing a critical role in the cardiovascular complications associated with metabolic diseases.

### 3.5. Gut Microbiota in MS

The human intestinal tract constitutes a nutrient-rich environment colonized by a highly diversified microbial species, collectively called “gut microbiota.” Endotoxin derived by microbial species triggers inflammation, leading to MS and contributing to OS. Growing evidence indicates that nutrients and environmental factors are tightly associated with ROS/RNS generation and gut microbiota and that nutritional factors such as certain natural compounds and nutraceuticals may ameliorate oxidative through the change of the microbiota [104]. Diets with elevated intake of high-energy foods have long been considered as a factor in the onset of MS and related diseases. Several studies on animals and humans have showed that consumption of diets rich in sugars and fat induces obesity, dyslipidemia, and insulin resistance, three of major MS components [105–109]. Nevertheless, the mechanisms by which these diets contribute to the pathogenesis of MS are not completely understood.

Studies on healthy adults have revealed that the gut microbial composition is highly host-specific. In fact, there is an elevated interindividual variation in terms of species, strain composition, and abundance and that this may contribute to variations in normal physiology and metabolism [109–113]. Moreover, it has been suggested that gut microbiota specificity is also determined by host genotype, age, sex, and health state [113, 114].

Gut microbiota participates in food digestion through two main catabolic pathways categorized as saccharolytic, with the production of the majority of short-chain fatty acid (SCFA), or proteolytic, which also induces SCFA formation, but leads to other co-metabolites potentially toxic predominantly renally cleared [115–117]. Furthermore, it has been reported that microbiota constitutes and regulates the intestinal mucosal barriers, controls nutrient uptake and metabolism, assists with maturation of immunological tissues, and prevents propagation of pathogenic microorganisms [117–120].

Studies on animal models and humans have also shown that changes in diet are associated with alterations in the composition and diversity of the community gut microbes and metabolic functions [109, 113, 121–128].

In particular, in animal models it has been shown that consumption of fructose, a major component of the Western-style diet, alters gut microbiota composition and induces several markers of the metabolic syndrome, inflammation, and OS. The fructose-rich diet also results in increased plasma levels of nonesterified fatty acid, plasmatic levels of bacterial lipopolysaccharide (LPS), and tumor necrosis factor (TNF-*α*) [125].

In humans, it has been suggested that both quantity and type of diet (fat or carbohydrate) can modify gut microbiota composition and also this can determine the development and rising incidence of obesity and MS [109, 122, 129–132].

Recent dietary interventions in subjects with high risk of developing CVD have shown that diets rich in fruit and vegetables improve cardiovascular health and impact on the gut microbiota [133–135]. In particular, the FLAVURS study showed that increasing the consumption of fruit and vegetables up to six 80 g portions daily caused a significant increase in total urinary flavonoids, vitamin C, and other phytochemicals [135–137].

Another strategy of modulating intestinal microbiota is by using of prebiotics. They are defined as live microorganisms used to reestablish an appropriate intestinal balance. They may potentially act through different mechanisms including pH modulation, antibacterial compound production, and competition with pathogens [113, 117, 138].

Typical prebiotics are mainly, although not all, dietary sources of fibers. In some studies, prebiotic administration is associated with both improved glycemic control and plasma lipid profiles [117, 128, 139, 140].

**Figure 3:**
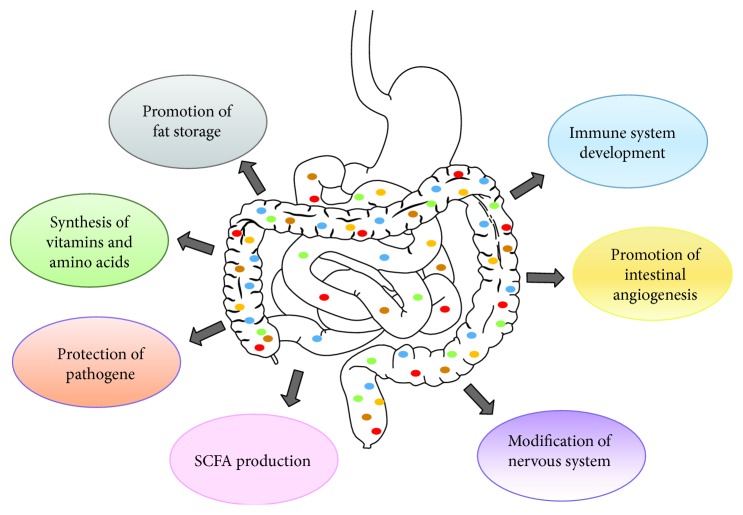
Main functions of bacteria in the gut. SCFAs: short-chain fatty acids.

It is possible that there may be other factors or mediators yet to be discovered that link gut microbiota to MS. More research is needed to elucidate the several mechanisms underlying diet–gut microbiota–host relationships that determine the development of MS. Main functions of bacteria in the gut are shown in Figure 3.

### 3.6. Antioxidant Systems and Antioxidant Therapy in MS

A significant decrease in the activity/expression of antioxidant systems has been found in MS patients. Cu, ZnSOD, Gpx, and CAT activity was lower in RBCs of obese women compared with cells of a normal weight group [141]. Interestingly, under inflammatory conditions, which characterize also MS, the expression of Gpx isoform 1 and MnSOD has been found downregulated as a consequence of the suppression of the transcription factor p53, leading to the increase in ROS formation and oxidative damage [142]. In addition to this, the decrease in p53 expression leads to adipose tissue inflammation, local insulin resistance, and cardiometabolic disorders [1, 143]. Furthermore, in addition to several biomarkers of OS (ox-LDL, 3-nitrotyrosine, monocyte O_2_^•^ formation, and NOX activity), low amounts of the antioxidant defense nuclear factor E2-related factor 2 (Nrf2) have been measured in blood of patients with MS. Nrf2 is an important antioxidant factor involved in controlling the nuclear transcription of antioxidant enzymes, such as GR, aldo-keto-reductase, heme-oxygenase-1, and *γ*-glutamyl-cysteine synthetase [144, 145].

Decreased levels of GSH have been measured in blood plasma as well as in RBCs [141] of MS patients, as well as reduced GSH/GSSG ratio animal models of obesity [50]. Interestingly, in addition to GSH depletion, a decreased activity of glucose-6-phosphate dehydrogenase, GR, and Gpx has been found in the serum of T2D patients [146]. As the main intracellular source of NADPH, a suitable activity of glucose-6-phosphate dehydrogenase is essential to maintain intracellular redox equilibrium. Therefore, the hyperglycemia-dependent inhibition of its activity in diabetic patients may have important repercussions on the entire NADPH-dependent antioxidant detoxifying system controlling the intracellular redox status. Moreover, under hyperglycemic conditions, the polyol pathway is the preferential way of glucose consumption [146]. The increased glucose flux in this pathway leads to (i) increased NADPH consumption in the conversion of glucose to sorbitol, (ii) increased NADH concentration, and (iii) decreased GSSG concentration linked to the downregulation of GR activity [146]. The increased NADH concentration could contribute to the increased OS linked to hyperglycemia, by favoring an overflux and recycling of NADH through the mitochondrial electron transport chain leading to ROS hyperproduction [146]. Moreover, under NADPH depletion conditions, the enzymes that use this cofactor as an electron donor cofactor, including NOXs and NOS, could also undergo to malfunction with important collateral effects such as vasoconstriction and platelet aggregation.

Finally, significantly elevated TrxR activity and Trx content have been measured in adipocytes present in subcutaneous tissue of obese people with metabolic disturbances, compared to metabolically healthy obese and control subjects [147]. The Trx/TrxR system could have a protective role in MS adipocytes and could represent an adaptive response to the altered redox environment [147, 148].

Therapeutic strategies aimed at reducing OS and enhancing antioxidant defense have been employed to restore redox balance and consequently to reduce cardiac dysfunction in the MS and related CDV diseases.

Some foods and some specific nutritional components have received special attention for the treatment of MS and related diseases. A diet with a higher total antioxidant capacity (TAC) in plasma has been associated with low OS low risk of abdominal obesity and diabetes mellitus. TAC is an indicator of diet quality defined as the sum of antioxidant activities of the pool of antioxidants present in a food [149]. Its concept was introduced because the evaluation of individual antioxidants does not reflect the antioxidant capacity of a whole diet or the interactions and synergistic effects of various antioxidants.

Mediterranean diet takes an adequate intake of fruits, vegetables, cereals, legume, fish, nut, red wine, and olive oil, which contain several nutritional components with anti-inflammatory and antioxidant properties [149, 150].

The PREDIMED study, a multicenter, randomized, controlled clinical trial, has shown that a Mediterranean diet increases TAC levels in subjects at high risk of cardiovascular disease [151].

The majority of clinical trials and studies in animal models of MS have been performed by using bioactive nutraceuticals from functional food, in particular vitamins (such as vitamins C and E), carotenoids, flavonoids, polyphenols, and selenium, which prevent the CVD in MS patients, although the underlying mechanisms were not fully elucidated for most of these compounds [152].

Vitamin C is an essential nutrient mainly found in fruits, especially citrus (lemon and orange), and vegetables (pepper and kale), whose benefits are associated with its antioxidant and anti-inflammatory properties. It produces its antioxidant effect primarily by quenching damaging free radicals and other reactive oxygen and nitrogen species and therefore preventing molecules such as ox-LDL [149].

Vitamin E, a fat-soluble phenolic compound contained in vegetable oils, is able to reduce OS and total cholesterol in patients with MS [153]. In diabetic patients, the supplementation of vitamins C and E, alone or together, significantly lowered hypertension, decreased hyperglycemia, and increased antioxidant activity, in particular the one of SOD and Gpx [153, 154]. Contrarily, in another study the combination of vitamins C and E did not affect body weights, glycated hemoglobin, LDL, or triglyceride amounts of patients with the MS or T2D [154]. On the other hand, vitamin C administration in an animal model contributed to lower hypertension by preventing the OS-mediated oxidation of the cofactor BH4 preventing NOS uncoupling [155]. With respect to the above vitamins, polyphenols and flavonoid**s** showed a greater efficacy in reducing metabolic and cardiac dysfunctions characterizing MS.

It has been reported that resveratrol (3,5,40-trihydroxystilbene), a phenolic compound mainly found in red grapes and derived products (red wine, grape juice), exerts antioxidant and anti-inflammatory activities, by inhibiting NF*κ*B signaling [156]. In an animal model, it protects the heart, coronary artery, and liver from dysfunctions generated by a high-fat and sugar-fed diet [157, 158]. In obese patients, it reduces insulin, glucose, and lipid concentrations in blood, as well as improving mitochondrial functions in skeletal muscle [159]. In a randomized, double-blind, placebo-controlled trial conducted on 50 MS patients treated for 8 weeks with red yeast rice-olive extract supplement, containing monacolin K and hydroxytyrosol, a reduction in total cholesterol, triacylglycerol, and ox-LDL has been demonstrated. No effects on plasmatic levels of MDA and 8-OHdG were detected [160].

Quercetin, a flavanol naturally present in vegetables, fruits, green tea, or red wine, reduces blood pressure and ox-LDL in blood plasma in overweight subjects with high cardiovascular disease risk [161]. Improved cardiac functions have been obtained by quercetin in animal models submitted to high-carbohydrate and high-fat diet [162].

Moreover, it has been reported that curcuminoid, sulforaphane, and epigallocatechin were effective in controlling hyperglycemia and improving the antioxidant response mediated by Nrf2 [163–165].

Curcuminoids are bioactive principles of the famous dietary spice, having a polyphenolic structure. A meta-analysis of randomized controlled trials shows that supplementation with curcuminoid-piperine combination significantly improves oxidative and inflammatory status in patients with MS [166].

### 3.7. Mitochondrial Dysfunction as Therapeutic Target

As reported above, mitochondria could contribute to the pathogenesis of MS with increased ROS production, alteration of energy metabolism, and dysfunction of the mitophagy process. Therefore, it is possible that it develops a strategy aimed at preventing mitochondrial dysfunction [167, 168]. The focus of these studies was to choose compounds with antioxidant capacity showing high mitochondrial affinity, such as SOD mimetics (pharmacological mimetics of antioxidant enzyme), coenzyme Q10 (a vitamin-like, lipid-soluble component of the mitochondrial electron-transport chain), and MitoQ (a triphenylphosphonium-conjugated derivative of coenzyme Q). Studies *in vitro* showed that SOD mimetics were effective in reducing ROS and restoring mitochondrial function [169], while in animal models of obesity, SOD mimetic and ONOO^−^ scavenger improved glucose tolerance [169]. Contrasting results were obtained in patients with MS, in which the addition of coenzyme Q10 to regular clinical therapy reduced diastolic dysfunction in children with cardiomyopathy [170], while the supplementation with the antioxidant was not sufficient to reduce hypertension in patients with the MS [170, 171]. In animal models of obesity, coenzyme Q10 supplementation reduced ROS formation and lipid peroxidation and ameliorated pressure parameters [172].

The use of MitoQ in animal models ameliorated cardiac dysfunction in rats subjected to ischemia/reperfusion [173] and decreased adiposity, hypercholesterolemia, and hypertriglyceridemia in high fat-fed mouse in models of MS [174]. No studies have yet been undertaken regarding the use MitoQ in clinical studies for human MS. The combined supplementation of MitoQ and MitoTEMPOL, another mitochondria-targeted antioxidant, improved mitochondrial activity and protected the coronary artery in rats subjected to ischemia/reperfusion [175].

## 4. Conclusions

Several lines of experimental and clinical evidences indicate that OS and redox imbalance play a pivotal role in the development of CDV linked to MS. The risk factors associated with MS, including obesity, diabetes, dyslipidemia, hypertension, and impaired glucose tolerance, are characterized by the persistence of OS-mediated chronic inflammatory conditions, as indicated by the occurrence of specific biomarkers in tissues from animals as well as from MS patients. OS may occur by multiple mechanisms, with prominent roles attributable to mitochondrial dysfunction, ROS/RNS-producing enzyme activation, and impairment of antioxidant system activity. Beyond its occurrence, it remains unclear whether OS may be the cause or consequence of MS. This is particularly important for mitochondrial-derived OS and dysfunction, which could be the main sources of oxidative damage and metabolic alterations in MS. Controversial findings have been obtained by using antioxidant therapies aimed at counteracting systemic as well as mitochondrial-derived OS. Other studies and clinical trials, by clarifying the mechanisms linking OS to MS, will allow developing specific and more suitable therapeutic approaches aimed not only at the control of oxidizing species formation but also at developing preventive strategies focused on limiting the occurrence and the progression of the cardiovascular risk factors.

Several factors link gut microbiota to MS. In fact, many data suggest that the gut microbiota has important physiological functions having a direct impact not only on host metabolism but also on gut mucosal barrier development and both local and systemic immune functions. Targeting gut microbiota composition or metabolic functions with natural and safe compounds, such as pro- or prebiotics, to promote a healthier profile might represent, indeed, a promising tool for prevention and treatment of obesity and correlated diseases.

Since OS has emerged as a central player in chronic metabolic diseases, it is imperative to (i) explore further the mechanisms that disrupt the normal equilibrium between oxidative and antioxidative processes; (ii) integrate the current therapies with various nutritional antioxidants such as flavonoids, arginine, vitamin C, vitamin E, carotenoids, resveratrol, and selenium capable of neutralizing OS; and (iii) prevent mitochondrial dysfunction to enhance defenses against OS.

## Figures and Tables

**Table 1:** Biomarkers of oxidative stress.

Biomarkers	Features
MDA (malondialdehyde)	Marker specific of omega-3 and omega-6 fatty acid peroxidation
HNE (4-hydroxy-2 nonenal)	Marker of arachidonic and linoleic acid oxidation
Sulfenic acid (SOH)	Marker of cysteine residue oxidation in specific proteins or enzymes (reversible)
Sulfinic (-SOOH) and sulfonic (-SOOOH) acids	Marker of cysteine residue oxidation in specific proteins or enzymes (irreversible)
Protein nitrosylation	Marker of cysteine residue modification induced by RNS (reversible)
Nitrated proteins	Irreversible tyrosine residue modification induced by RNS
Carbamylated proteins	Generated following the attack of the ^•^OH radical to the residues of proline, lysine, and arginine
Oxidized LDL	Generated by LDL oxidation with ROS, RNS, and carbon-centered radicals

## Abbreviations

8oxodG: 8-Oxo-2′-deoxyguanosine
AGE: Advanced glycation end products
ATP: Adenosine triphosphate
BH4: (6R)-5,6,7,8-Tetrahydro-L-biopterin
CAT: Catalase
COX: Cyclooxygenase
CuZnSOD: Copper-zinc superoxide dismutase
CVD: Cardiovascular diseases
DUOX: Dual oxidases
EC-SOD: Extracellular superoxide dismutase
eNOS: Endothelial nitric oxide synthase
EPR: Electron paramagnetic resonance
FFAs: Free fatty acids
Gl-LDL: Glycated low-density lipoprotein
Gpx: Glutathione peroxidase
GR: Glutathione reductase
Grx: Glutaredoxin
GSH: Reduced glutathione
GSSG: Oxidized glutathione
HDL: High-density lipoprotein
HNE: 4-Hydroxy-2-nonenal
IL-6: Interleukin-6
iNOS: Inducible nitric oxide synthase
LDL: Low-density lipoprotein
LOOH: Lipid hydroperoxide
LOX: Lipoxygenase
LPS: Lipopolysaccharide
MCP-1: Monocyte chemoattractant protein-1
MDA: Malondialdehyde
MnSOD: Manganese superoxide dismutase
MPO: Myeloperoxidase
MS: Metabolic syndrome
NF*κ*B: Nuclear factor kappa-light-chain-enhancer of activated B cells
nNOS: Neuronal nitric oxide synthase
NOS: Nitric oxide synthase
NOXs: NADPH oxidase isoforms
Nrf2: Nuclear factor E2-related factor 2
OS: Oxidative stress
ox-LDL: Oxidized low-density lipoprotein
PGC-1a: Proliferator-activated receptor gamma coactivator 1a
PKC: Protein kinase C
Prx: Peroxiredoxin
PUFAs: Polyunsaturated fatty acids
RAGE: Soluble receptor of AGE
RBCs: Red blood cells
RNS: Reactive nitrogen species
RO^•^: Alkoxyl radical
ROO^•^: Peroxyl radicals
ROS: Reactive oxygen species
RS^•^: Thiyl radicals
SCFA: Short-chain fatty acids
SOD: Superoxide dismutase
T2D: Type 2 diabetes
TAC: Total antioxidant capacity
TNF-*α*: Tumor necrosis factor
TNF-*α*: Tumor necrosis factor alpha
TO^•^: Tocopheroxyl radicals
Trx: Thioredoxin
TrxR: Thioredoxin reductase.
